# OBP-Mediated Molecular Mechanism Underlying the Olfactory Repellent Effect of *Mosla chinensis* Essential Oil Against *Culex quinquefasciatus*

**DOI:** 10.3390/genes17060707

**Published:** 2026-06-19

**Authors:** Jinfeng Xiong, Rui Ma, Ya Wu, Guoxiu Wang, Hui Ai

**Affiliations:** 1 Key Laboratory of Pesticide & Chemical Biology of Ministry of Education, School of Life Sciences, Central China Normal University, Wuhan 430079, China; 2 Academy of Frontier Interdisciplinary Research, Central China Normal University, Wuhan 430079, China; 3 Research Center for Territorial Spatial Conservation, Utilization and Computational Governance, Central China Normal University, Wuhan 430079, China

**Keywords:** *Culex quinquefasciatus*, *Mosla chinensis*, plant essential oil, odorant-binding protein, binding capacity

## Abstract

Background/Objectives: Mosquitoes, including *Culex quinquefasciatus* and *Aedes aegypti*, are important vectors of dengue fever, Zika virus, West Nile virus, Japanese encephalitis virus, Eastern equine encephalitis virus, etc. Biological control has always been urgent in mosquito prevention due to resistance developing to synthetic insecticides and environmental toxicity by insecticides. Methods: The leaf essential oil of *Mosla. chinensis* was isolated, and major components were identified via GC-MS, followed by olfactory behavior assays to evaluate its repellent activity against *C. quinquefasciatus*. Additionally, the *odorant-binding protein 1* and *odorant-binding protein 2* (*CquiOBP1-2*) genes were prokaryotically expressed, and their fluorescence competitive binding activities with the active components of essential oils were examined. Results: The bioassays indicated this essential oil greatly repels *C. quinquefasciatus*, which will significantly protect people against vector-borne diseases. In the fluorescence competitive binding experiments, the CquiOBP1-2 proteins exhibit great binding capacities to volatile components, including Citronellal, Citronellol, Geraniol, Limonene and Isopulegol. Furthermore, the behavioral experimental results also indicate that the mixture of these five ligand compounds has an obvious repellent effect on mosquitoes, highlighting that they may be applied as potential mosquito repellent agents. Moreover, molecular docking and site-directed mutation analysis further confirm Phe123 and Gln77 are both key amino acid residues of CquiOBP1-2 proteins involved in the olfactory recognition of repellent ligand compounds from *M. chinensis* essential oil. Conclusions: The behavioral experimental verification and the exploration of olfactory molecular mechanisms are helpful to promote the biological control of plant essential oils in mosquito pests.

## 1. Introduction

*C. quinquefasciatus*, a nocturnal domestic mosquito mainly occurring in tropical and subtropical regions, is a primary vector of West Nile virus, Japanese encephalitis virus, Eastern equine encephalitis virus, St. Louis encephalitis virus and lymphatic filariasis [1]. Vector control is the most successful way for reducing incidences, and chemical insecticides are still the most effective against mosquitoes [2]. Organophosphorus insecticides are usually used as larvicides for outdoor mosquito control, while pyrethroid insecticides are currently used for indoor adult control [3,4]. However, the heavy and long-term use of insecticides in public health has promoted the development of insecticide resistance [5,6]. Moreover, the extensive use of chemical insecticides not only easily polluted the ecological environment but also caused damage to non-target organisms such as pollinators, bees and butterflies [7]. Thus, alternative vector control strategies such as insect growth regulators (IGRs), microbial pesticides, plant essential oils, and a large number of highly efficient, low-toxicity biological mosquito repellents are urgently needed and have become important choices [8,9].

Plant essential oils can have bacteriostatic, insecticidal, antioxidant or other activities and are widely used in medicine, pesticides, feed additives, food preservation, etc. [10]. The essential oils obtained from different plants have also been widely reported in the control of various mosquitoes. For instance, Samir et al. isolated three plant essential oils from *Artemisia monosperma*, *Origanum vulgare* and *Schinus mole* and evaluated their toxic and developmental properties against *Culex pipiens* [11]. They found essential oils can significantly prolong the larval and pupal stages and greatly shorten the adult periods of *C. pipiens* [12]. The *Neolitsea ellipsoidea* essential oil was rich in (E)-β-ocimene (87.6%) and exhibited excellent larvicidal activity against *Ae. aegypti*, which should be considered for utilization as alternative agents for controlling mosquito populations [13]. However, there were few reports on the olfactory repellent molecular mechanism of plant essential oil, which was of great significance for their extensive application and precise prevention and control in the field [9,10].

*M. chinensis* is a type of traditional medicinal plant and is widely distributed in China, Vietnam, Thailand, Laos, Japan, Malaysia and other Southeast Asian countries. In this study, we isolated the leaf essential oil of *M. chinensis* and identified its main components by the GC-MS technology. Subsequently, the repellent activities of essential oil against *C. quinquefasciatus* were tested by the olfactory behavior experiments. Also, the *CquiOBP1-2* genes were cloned from the antennae of *C. quinquefasciatus* and purified through prokaryotic expression and affinity chromatography. And the fluorescence competitive binding assay was used to determine the olfactory repellent molecular mechanism of ligand compounds from *M. chinensis* essential oil against mosquitoes. These experimental studies will not only help to elucidate the molecular mechanism of mosquito repellency but also contribute to comprehensive prevention and control of *C. quinquefasciatus* in the field.

## 2. Materials and Methods

### 2.1. Identification of Plant Essential Oil and Repellent Assays Against Mosquitoes

The *M. chinensis* fresh leaf was collected, and plant essential oil was isolated through a Clevenger-type apparatus [14]. The gas chromatography-mass spectrometry (GC-MS, Agilent 5977C, Santa Clara, CA, USA) technique was applied to determine the detailed composition of *M. chinensis* essential oil. The GC-MS analysis was analyzed on a chromatographic column (30 m × 0.25 mm, 0.25 μm) with helium (1.0 mL/min), a 60–280 °C ramp at 5 °C/min, EI at 70 eV and full scan (*m*/*z* 30–550). The *C. quinquefasciatus* adults were randomly separated into different experimental groups (twenty-five each) and kept in the mosquito cage (30 × 30 × 30 cm). Different concentrations of *M. chinensis* essential oils (1.25 μL, 2.5 μL, 5 μL, 10 μL, 20 μL) were used in behavioral experiments. The plant essential oil was applied on the ventral part of volunteers’ forearms, which were then inserted into a cage for repellent tests according to the experimental method described by Nasrul et al. [15].

### 2.2. Sequence Analysis and Prokaryotic Expression of Olfactory Proteins

The amino acid sequences of *C. quinquefasciatus* odorant-binding protein 1 and odorant-binding protein 2 proteins (CquiOBP1-2) and other mosquitoes’ OBPs were obtained from the NCBI database. The DNAMAN software (v9.0) was used for their amino acid multiple alignments, and N-terminal signal peptides were predicted by the SignalP4.1 Server (https://services.healthtech.dtu.dk/services/SignalP-6.0/ accessed on 7 June 2026). According to the signal peptide analysis results, prokaryotic expression primers of *CquiOBP1* (Genbank number: AAL86413.1) and *CquiOBP2* (Genebank number: FJ947084.1) genes were designed, and recombinant plasmids PET32A-CquiOBP1 and PET32A-CquiOBP2 with His-tags were constructed (without signal peptides) (Table 1). After the PET32a-CquiOBP1-2 was transformed into the DH5α strain, the recombinant plasmid was extracted again and further transformed into competent *Escherichia coli* BL21 (DE3) cells. The CquiOBP1-2 genes were induced by 0.5 mmol/L IPTG for 4 h and expressed in the *E. coli* prokaryotic expression system. Subsequently, the bacterial suspension was collected by centrifugation at 5000 rpm for 3 min. Then, the *E. coli* suspension was suspended in PBS buffer solution, and the ultrasonic crushing was used to separate the bacterial suspension after induction. The protein SDS-PAGE analysis was used for detection, and CquiOBP1-2 proteins were stored in −80 °C refrigerator until use.

### 2.3. Ligand Binding Experiment of CquiOBP1-2 Proteins with M. chinensis Essential Oil

After the recombinant CquiOBP1-2 proteins were purified, the ligand binding experiment of CquiOBP1-2 proteins with the volatile repellent compounds was carried out according to our previous experimental method of Mao et al. [16]. The bindings of N-phenyl-1-naphthylamine (1-NPN) and potential candidate ligands from *M. haplocalyx* essential oil were measured by a Hitachi F-4500 fluorescence spectrophotometer (Hitachi, Ltd., Tokyo, Japan). The scanning emission wavelength range was 370–500 nm, and the excitation wavelength was 337 nm. The binding capacity curve of OBP proteins with 1-NPN was measured by adding 1-NPN with a gradient of increasing concentration to a 2 μmol/L recombinant OBP protein solution (dissolved in Tris-HCl, pH 7.4), and then the binding value to the protein was recorded, and the experiment was repeated three times. The binding ability of OBP protein with the ligand is determined by adding OBP protein and 1-NPN (the final concentration is 2 μmol/L) in a quartz dish, gradually adding the ligand to a final concentration (from 4 μmol/L to 48 μmol/L). The competitive dissociation constant *Ki* was calculated using the Cheng-Prusoff equation: *K_i_* (*K_i_* = IC_50_/(1 + [1-NPN]/*K_1-NPN_*) [16]. IC_50_ is the concentration when the ligand replaces 50% of probe 1-NPN; [1-NPN] is the concentration of unbound 1-NPN; *K_1-NPN_* is the dissociation constant of the OBP protein and 1-NPN. After the potential repellent ligands were screened out by the fluorescence competitive binding experiments, the mosquito repellent tests were measured according to the experimental method described by Nasrul et al. [15]. When a volatile compound at a certain concentration in the binding curve is able to displace 50% of 1-NPN from the target protein/1-NPN complex, the compound is considered to have binding activity.

### 2.4. Homology Modeling and Molecular Docking

The CquiOBP1-2 protein sequences were analyzed in the SWISS-MODEL server (http://swissmodel.expasy.org/) and PyMOL software (v3.0). We found that the CquiOBP1 protein had a definite crystalline protein structure (3ogn.1), while the CquiOBP2 protein shared high similarity (67.21%) with the homologous *Ae. aegypti* OBP1 protein (crystalline structure number: 3k1e.1). The predicted protein structure model of the CquiOBP2 protein was built by homology modeling, and the potential mosquito repellent ligand structure was obtained from the ZINC compounds database (https://zinc.docking.org/). The reliability of the protein structural models was verified using validation modules from the SAVES 6.0 online analysis platform (https://saves.mbi.ucla.edu/). The best model with the most favorable geometric properties was selected for molecular docking. Subsequently, the AutoDock (v4.2.6) Vina program was used to find hydrogen bonding sites between CquiOBP1-2 proteins and potential repellent ligands.

### 2.5. Site-Directed Mutation Analysis and Binding Affinities of Mutant OBP Proteins

The site-directed mutation analysis of CquiOBP1-2 proteins was performed according to the experimental protocol described by the fast mutagenesis system kit (Transgen, Beijing, China). After the mutant plasmid was detected by sequencing, it was transformed into BL21 (DE3) competent cells for prokaryotic expression. The experimental methods of vector construction and recombinant protein expression were the same as the above prokaryotic expression and purification method. The CquiOBP1-2 mutant proteins were purified and used for the verification of fluorescence competitive binding experiments with the potential mosquito repellent ligands. The effective repellent compounds will be identified by site-directed mutation, fluorescence competition affinity assays, and the repellent behavior test of mosquitoes.

## 3. Results

### 3.1. Chemical Composition Identification of Essential Oil and Repellent Activity

**Figure 1:**
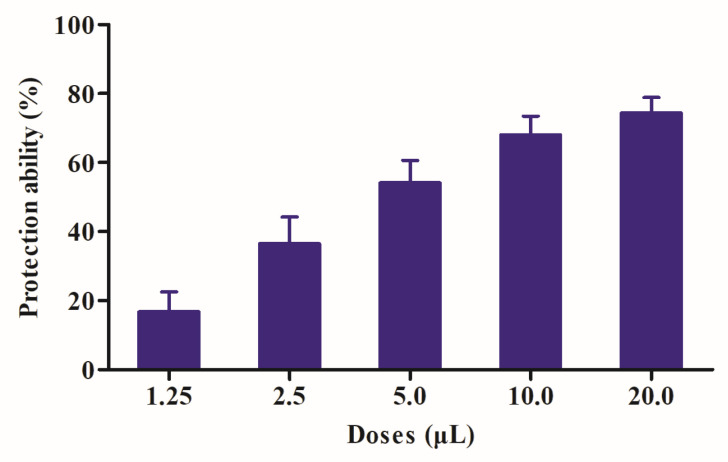
Mosquito repellent activity of essential oil from *Mosla chinensis* against *C. quinquefasciatus* adults.

GC-MS analysis results demonstrated that there were 15 compounds with relatively high content in *M. chinensis* essential oil (Table 2). The major six compounds with high content were Citronellal (31.46%), Citronellol (12.74%), Geraniol (18.50%) Citronellol acetate (4.15%), Limonene (4.22%), and Geranyl acetate (3.47%), respectively. And other compounds with more than 1% content were Cyclohexane, 1,6-Cyclodecadiene, Isopulegol, Muurolene and Cadinol. These eleven kinds of chemical components may play a key role in the repellent activity of *M. chinensis*. Figure 1 shows the *M. chinensis* essential oil significantly protected the arm of volunteers, especially when used at higher concentrations.

### 3.2. Sequence Analysis and Recombinant Expression of CquiOBP1-2 Proteins

**Figure 2:**
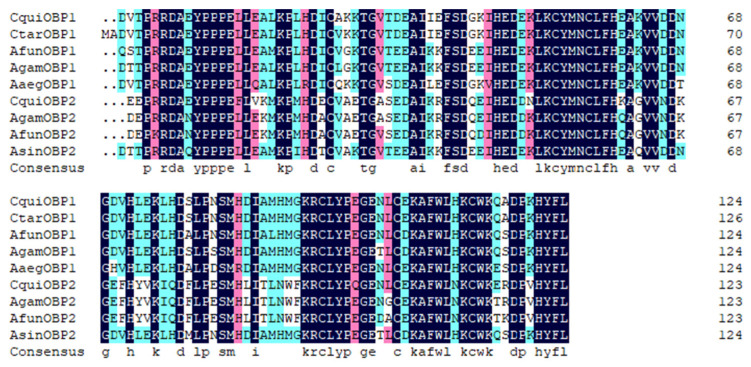
Multiple sequence alignment of CquiOBP1-2 with other mosquito OBP proteins. CtarOBP1 (AY189987.1), AfunOBP1 (HM436669.1), AgamOBP1 (AF437884.1), AaegOBP1 (AY189225.1), AgamOBP2 (AF437886.1), AfunOBP2 (HM436670.1), AsinOBP2 (KJ958382.1).

**Figure 3:**
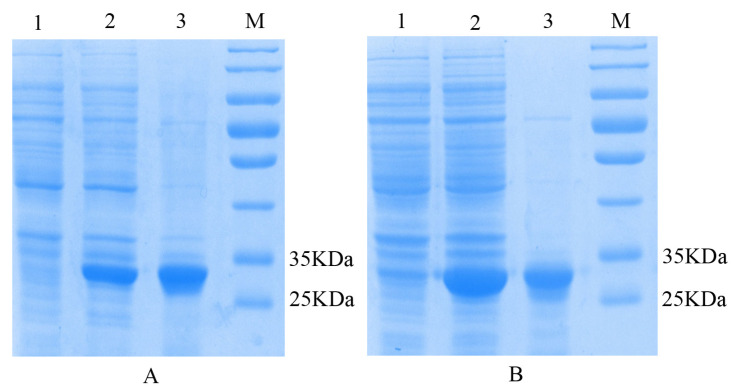
SDS-PAGE electrophoretic analysis of olfactory proteins. (**A**) CquiOBP1; (**B**) CquiOBP2. Lane 1-noninduced *E. coli* OBP, Lane 2-induced *E. coli* OBP, Lane 3-purified OBP, Lane M-marker protein.

Amino acid multiple alignments of a variety of mosquitoes’ OBP proteins indicated that the CquiOBP1 protein shared high identities at the amino acid level with AaegOBP1 (87.40%), AfunOBP1 (87.40%), AgamOBP1 (88.98%) and CtarOBP1 (97.64%), respectively (Figure 2). The CquiOBP2 protein was similar to AgamOBP2, AfunOBP2 and AsinOBP2 with identity values of 89.76%, 85.83% and 64.57%, respectively (Figure 2). In the *E. coli* prokaryotic expression system, the *CquiOBP1* and *CquiOBP2* genes were induced to express in large quantities and obtained by Ni-NTA resin affinity chromatography after ultrasonication. The SDS-PAGE gel electrophoresis analysis result demonstrated that the CquiOBP1-2 proteins were purified from the supernatant and showed high purity (Figure 3). The predicted molecular weight of the target proteins was consistent with the molecular weight determined by electrophoresis.

### 3.3. Ligand Binding Activities of CquiOBP1-2 Proteins

**Figure 4:**
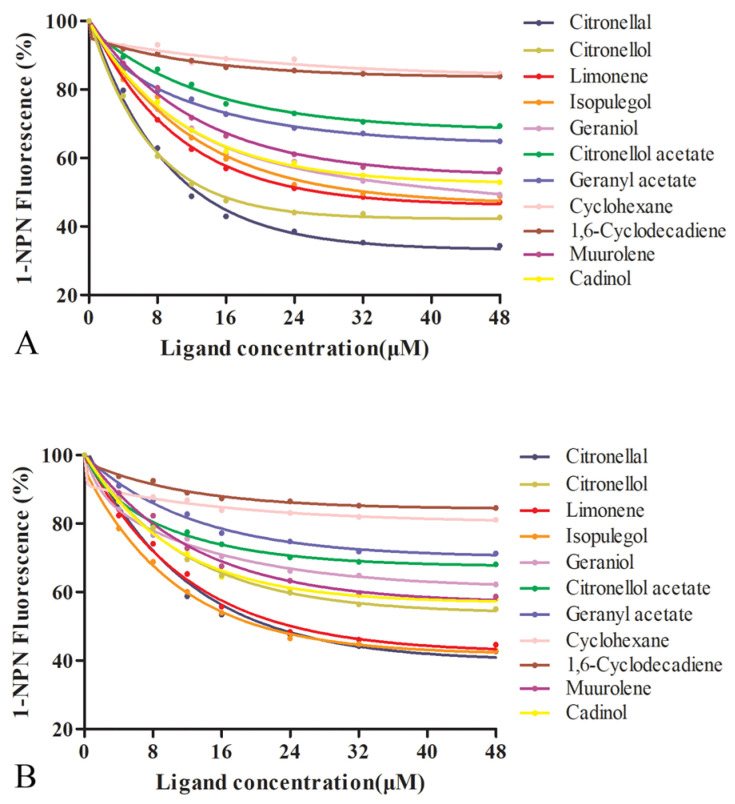
Competitive binding curves of repellent ligands to CquiOBP1-2 proteins in the ligand-binding experiments ((**A**) CquiOBP1 and (**B**) CquiOBP2).

The Scatchard plots and binding curves suggested that the binding capacities of CquiOBP1-2 proteins and the fluorescence probe increased with increasing concentration of 1-NPN (Figure S1). The fluorescence competitive binding results indicated that only 5 kinds of odor molecules could replace fluorescent probe 1-NPN by more than 50% from the recombinant CquiOBP1 protein solution, indicating that CquiOBP1 had a great odor binding spectrum (Figure 4A). The CquiOBP1 protein showed the best binding affinity to Citronellal and Citronellol, with the Ki values (the calculated inhibition constants) of 12.35 μM and 13.88 μM (Table 3). CquiOBP1 also exhibited obvious binding activities to Geraniol, Limonene and Isopulegol. with the *Ki* values of 26.64 uM, 19.42 μM and 21.83 μM, respectively (Figure 4A and Table 3). Additionally, CquiOBP2 protein was the most sensitive to Isopulegol, Citronellal and Limonene, whose *Ki* values were 17.51 μM, 18.07 μM and 19.56 μM (Figure 4B and Table 3).

### 3.4. Homology Modeling and Molecular Docking and Site-Directed Mutation Analysis

**Figure 5:**
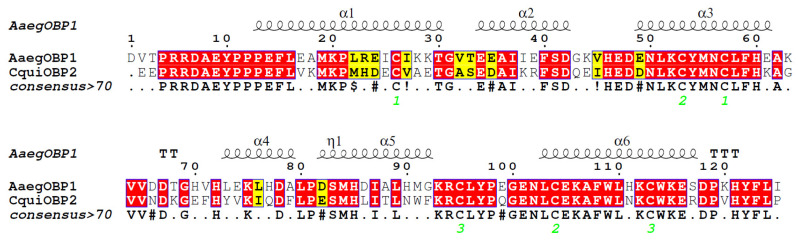
Sequence alignment of CquiOBP2 and *Ae. aegypti* OBP1 proteins. Conserved residues are highlighted in white letters with a red background. Six conserved residues are labeled by a pentagram. The disulfide bridges are numbered 1 to 3. α-helices are displayed as squiggles.

**Figure 6:**
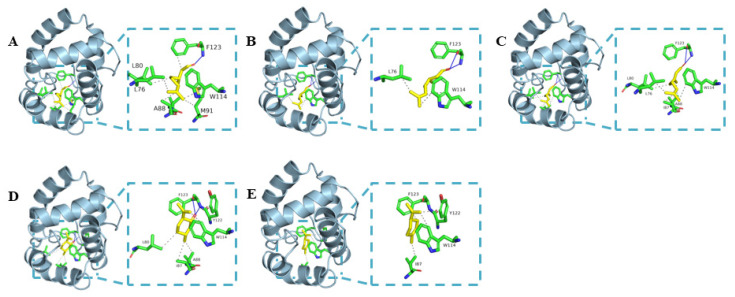
Molecular docking of the CquiOBP1 protein to five ligand components from *Mosla chinensis* essential oil. (**A**–**E**) Citronellal, Citronellol, Geraniol, Isopulegol, Limonene.

**Figure 7:**
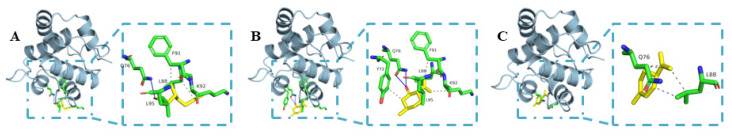
Molecular docking of the CquiOBP2 protein to three ligand components from *M. chinensis* essential oil. (**A**–**C**) Citronellal, Isopulegol, Limonene.

Homology modeling and molecular docking were applied to analyze potential key binding sites of CquiOBP1-2 proteins with volatile repellent ligands. The crystal structure of the CquiOBP1 protein was found in the SWISS-MODEL server and the *Ae. aegypti* OBP1 (3k1e.1) protein was chosen as the template of the CquiOBP2 protein by the amino acid sequences comparison (Figure 5). Subsequently, the 3D structures of the CquiOBP2 protein were also constructed by SWISS-MODEL, and volatile repellent ligands with great binding capacities were docked into the binding pockets of the CquiOBP1 and CquiOBP2 protein models (Figure 6 and Figure 7). As shown in Table 4, the key amino acid binding sites of CquiOBP1 and CquiOBP2 proteins with ligands were Phe123 (phenylalanine, F123) and Gln77 (glutamine, Q77), respectively.

### 3.5. Site-Directed Mutation and Validation of Fluorescence Competitive Binding Affinities

**Figure 8:**
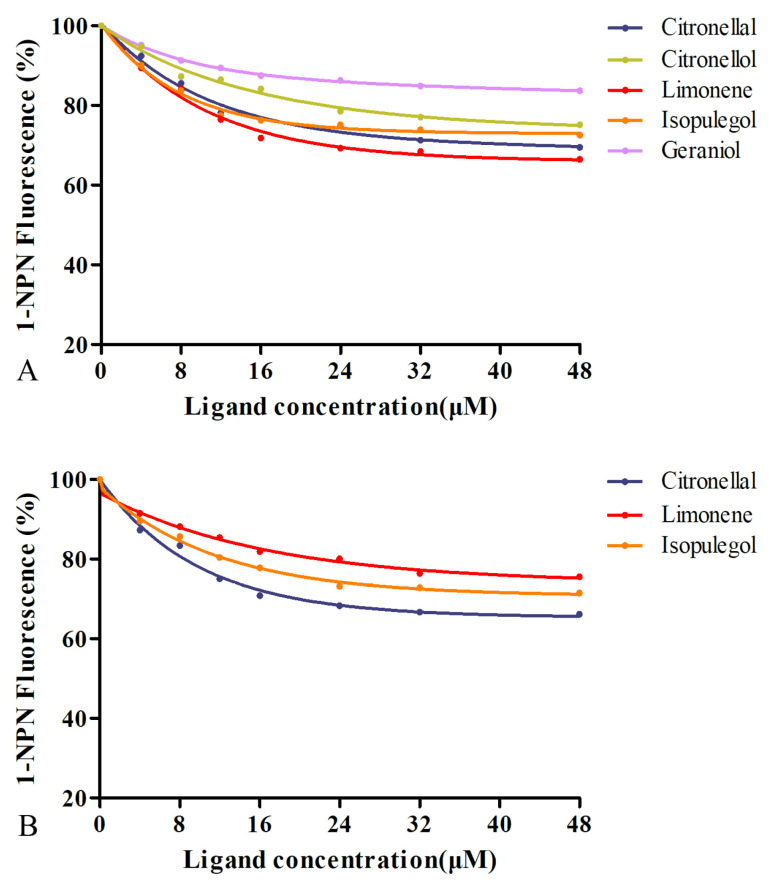
Competitive binding curves of repellent ligands to mutant CquiOBP1-2 proteins in the ligand-binding experiments ((**A**) CquiOBP1-F123A and (**B**) CquiOBP2-Q77A).

After site-directed mutagenesis, both key amino acid residues (F123 and Q77) of CquiOBP1 and CquiOBP2 proteins were substituted with Ala (alanine, A), respectively. Subsequently, two kinds of mutants, F123A and Q77A, were measured for their binding properties with ligand compounds using the fluorescence probe 1-NPN. As shown in Figure S2, the SDS-PAGE analysis results of mutant CquiOBP1-2 proteins suggested recombinant target proteins were greatly purified from the prokaryotic expression system. As shown in Figure S3, the binding capacities of mutant OBP proteins and 1-NPN increased with increasing concentration of the fluorescence probe. In present binding assays, compared with wild-type CquiOBP1 protein, mutant CquiOBP1-F123A lost its capacity to bind ligand compounds Citronellal, Citronellol, Geraniol, Limonene and Isopulegol (Figure 8A), suggesting that Phe123 was an important amino acid residue of CquiOBP1 involved in the binding to ligand compounds. And the binding efficiency of mutant CquiOBP2-Q77A to the three ligand compounds, Citronellal, Limonene and Isopulegol also significantly decreased and did not displace 50% of 1-NPN from the CquiOBP2-Q77A/1-NPN complex solution (Figure 8B), revealing that Gln77 was one of the key amino acid residues of the CquiOBP2 protein for binding ligand compounds.

### 3.6. Behavioral Test of the Identified Effective Repellent Compounds

**Figure 9:**
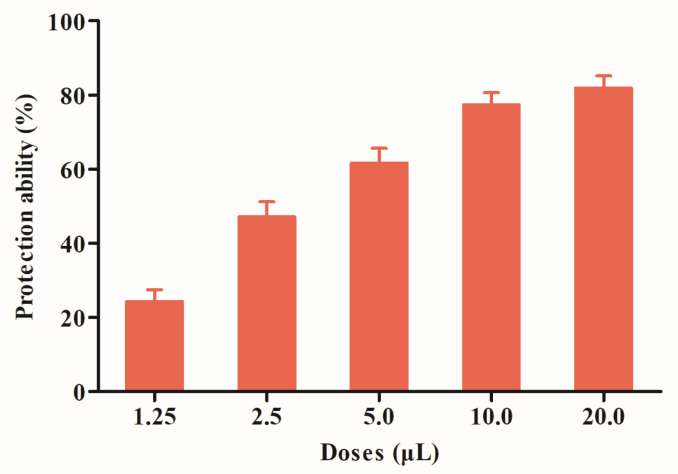
Repellent behavioral validation of effective compounds obtained from *Mosla chinensis* against mosquitoes.

Based on the above results of molecular docking and site-directed mutation analysis, five effective repellent compounds, including Citronellal, Citronellol, Geraniol, Limonene and Isopulegol, were obtained from *M. chinensis* essential oil. In order to investigate repellent abilities of these five identified compounds against mosquitoes, the repellent experiment was again used in the behavior experiments of adult *C. quinquefasciatu*. As shown in Figure 9, the behavioral test results indicated that the mixture of the five ligand compounds had an obvious repellent effect on *C. quinquefasciatu*.

## 4. Discussion

Japanese encephalitis is a mosquito-borne zoonotic disease that is endemic to Southeast Asia and China; 68,000 cases and 10,000 deaths are estimated each year globally [17,18]. Vectors play an important role in the transmission of arboviruses, and *C. quinquefasciatus* is one of the primary vectors for Japanese encephalitis [19]. Control of mosquito vectors has long been a serious part of the global policy to eliminate mosquito-borne diseases. At present, due to the abuse of chemical insecticides, many mosquitoes have developed strong resistance to insecticides and easily pollute the environment. Natural repellents have many advantages, such as low toxicity, a fresh smell, no drug residue, easy degradation and so on. Therefore, natural non-toxic insecticides and repellents, including plant essential oils, are becoming the first choice for insect prevention and mosquito repellent. *M. chinensis* was widely distributed in Asian countries, and it has been recorded as a traditional medicine to treat many diseases of fever, dysentery and other digestion disorders [20]. The present experimental results also show that the *M. chinensis* essential oil contained rich repellent compounds and could play an obvious repellent effect against mosquito adults. The bioassay results of *Etlingera elatior* essential oil also exhibit similar repellent effects and oviposition deterrent activities against *Ae. aegypti* females, suggesting that plant essential oils may play an important role in the repellent or oviposition site-seeking behavior of pests [21,22]. However, the olfactory molecular mechanism of the repellent activities of these essential oils remains unclear.

In this study, the fluorescence competitive binding analysis indicated that CquiOBP1 protein could effectively bind five kinds of volatile ligand compounds from *M. chinensis* essential oil, highlighting that these five volatiles may have potential mosquito repellent activities. Among all compounds, the CquiOBP1 protein showed the highest binding capacities to Citronellal and Citronellol. Moreover, Gillij et al. found that Citronellal and Citronellol belong to the terpene components, and behavioral experimental results also showed their obvious repelling effect against adult mosquitoes, which are associated with insect repellent properties [23]. Among the ligand compounds, Citronellal, Limonene and Isopulegol also have great binding abilities with the CquiOBP2 protein, suggesting that these three chemical molecules may serve an important function in the repellent activities of *M. chinensis* essential oil. Similar studies have been reported on the function of other mosquito olfactory proteins. For instance, *Anopheles sinensis* OBP1 protein was also able to bind to N, N-diethyl-m-toluamide (DEET), which provided more basic information for olfactory chemical control of mosquitoes [24].

Based on the analysis results of the binding characteristics of olfactory proteins and ligands, the chemical molecules with strong binding abilities were selected for molecular docking. It was found that the binding of CquiOBP1 and CquiOBP2 to ligands mainly depended on the hydrophobic effect and hydrogen bond between the key amino acid sites (OBP1-F123A and OBP2-Q77A) and ligand molecules. The site-directed mutation analysis also further confirmed the chemical interaction between the five ligand compounds and mosquito olfactory proteins. Compared with the pre-mutant protein, the binding abilities of the mutant OBP proteins to the chemical components of the essential oil were significantly decreased. Moreover, the behavioral test results suggested that these five compounds had obvious repellent activities against *C. quinquefasciatus*, indicating that they had a great field control effect on adult mosquitoes. Citronellal, Citronellol and DEET compounds have also been reported to repel other mosquitoes, highlighting that many plant essential oils could be used in integrated mosquito control. Subsequently, further functional studies of olfactory genes through RNAi or gene editing, electrophysiology, behavioral and structural biology will provide more theoretical and scientific bases for the comprehensive control of *C. quinquefasciatus* so as to avoid the toxic damage of mosquitoes to human beings.

## Figures and Tables

**Table 1:** Primers used in the experiments.

Primer Name	Sequence (5′-3′)
OBP1-BamHIF	CGCGGATCCGACGTTACACCCCGTCG
OBP1-Xho1R	CCGCTCGAGTTAAACCAGGAAATAATGCTTT
OBP2-BamHIF	CGCGGATCCGAGGAACCGAGGCGAGA
OBP2-Xho1R	CCGCTCGAGTTAGGGCAAAAAGTAGTGCAC

**Table 2:** The chemical composition of *Mosla chinensis* essential oil.

Compounds	Abundance (%)
Linalool	0.79
Citronellal	31.46
Citronellol	12.74
Geraniol	18.50
α-Citral	0.56
Citronellol acetate	4.15
Eugenol	0.97
Geranyl acetate	3.47
Cyclohexane	2.46
Naphthalene	0.67
1,6-Cyclodecadiene	3.40
Limonene	4.22
Isopulegol	1.55
Muurolene	1.48
Cadinol	1.21

**Table 3:** The binding constants of different ligands with the CquiOBP1-2 protein.

No.	Compounds	CquiOBP1		CquiOBP2	
		IC50 (μM)	*Ki* (μM)	IC50 (μM)	*Ki* (μM)
1	Citronellal	14.54	12.35	20.37	18.07
2	Citronellol	16.37	13.88	-	-
3	Geraniol	31.47	26.64	-	-
4	Citronellol acetate	-	-	-	-
5	Geranyl acetate	-	-	-	-
6	Cyclohexane	-	-	-	-
7	1,6-Cyclodecadiene	-	-	-	-
8	Limonene	22.82	19.42	21.97	19.56
9	Isopulegol	25.61	21.83	19.65	17.51
10	Muurolene	-	-	-	-
11	Cadinol	-	-	-	-

Note: ‘-’ indicates no binding.

**Table 4:** The docking results of CquiOBP1-2 proteins with different ligands.

Ligand	Different Residues Within 4.0 Å		Residues Forming H-Bond with Ligand	
	CquiOBP1	CquiOBP2	CquiOBP1	CquiOBP2
Citronellal	Leu76, Leu80, Ile87, Ala88 Met91, Trp114, Phe123	Leu88, Phe91, Lys92, Leu95	Phe123	Gln77
Citronellol	Leu76, Trp114		Phe123	-
Geraniol	Leu76, Leu80, Ile87, Ala88, Trp114		Phe123	-
Limonene	Ile87, Trp114, Tyr122, Phe123	Gln77, Leu88	-	-
Isopulegol	Leu80, Ile87, Ala88, Trp114, Tyr122	Tyr72, Leu88, Phe91, Lys92, Leu95	Phe123	Gln77

## Data Availability

The original contributions presented in this study are included in the article/Supplementary Material. Further inquiries can be directed to the corresponding author.

## Supplementary Materials

The following supporting information can be downloaded at https://www.mdpi.com/article/10.3390/genes17060707/s1, Figure S1: Binding curve and relative scatchard plot in the ligand-binding experiments of CquiOBP1-2 proteins; Figure S2: SDS-PAGE electrophoretic analysis of mutant CquiOBP1-F123A and CquiOBP2-Q76A proteins. Lane 1—noninduced *E. coli* OBP, Lane 2—induced *E. coli* OBP, Lane 3—purified OBP, Lane M—Marker protein. Figure S3: Binding curve and relative scatchard plot in the ligand-binding experiments of CquiOBP1-2 proteins (A, CquiOBP1 and B, CquiOBP2).
