# Supplementary material for: OBP-Mediated Molecular Mechanism Underlying the Olfactory Repellent Effect of Mosla chinensis Essential Oil Against Culex quinquefasciatus
Source: Genes (Basel). 2026 Jun 19;17(6):707. doi: 10.3390/genes17060707 (PMC13299321; doi:10.3390/genes17060707)
Supplement: Supplementary file 1 [file genes-17-00707-s001.zip › genes-4227890-supplementary.pdf]

Supplementary figure documents

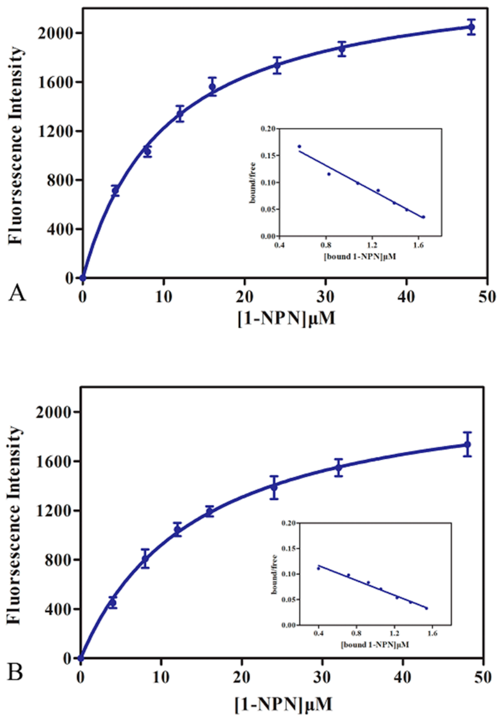

Figure S1. Binding curve and relative scatchard plot in the ligand-binding experiments of CquiOBP1-2 proteins (A, CquiOBP1 and B, CquiOBP2).

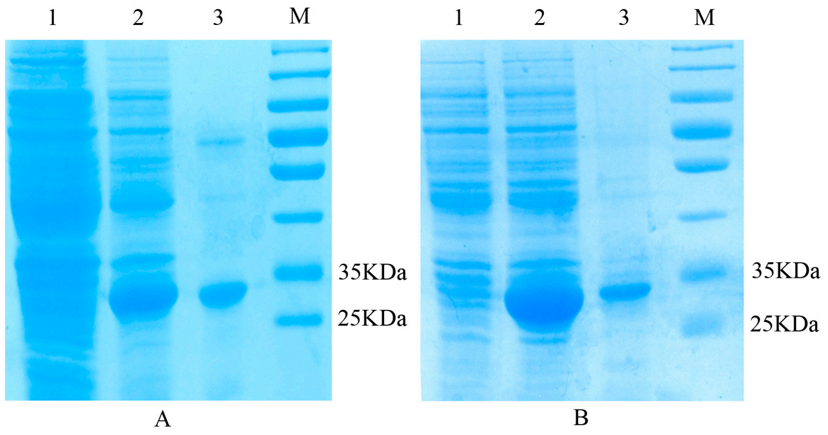

Figure S2. SDS-PAGE electrophoretic analysis of mutant CquiOBP1-F123A and CquiOBP2-Q76A proteins. Lane 1 - noninduced *E. coli* OBP, Lane 2 - induced *E. coli* OBP, Lane 3 - purified OBP, Lane M - Marker protein.

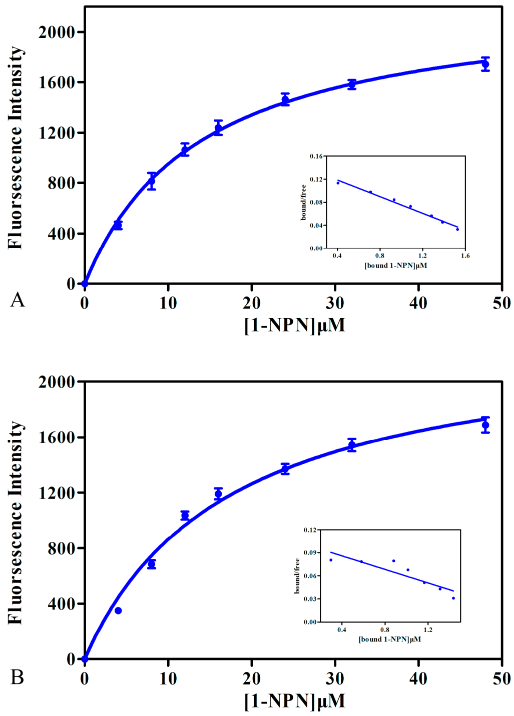

Figure S3. Binding curve and relative scatchard plot in the ligand-binding experiments of CquiOBP1-2 proteins (A, CquiOBP1 and B, CquiOBP2).
